# *Palisada yatsenii* sp. nov. (Ceramiales, Rhodophyta), a New Prostrate Red Alga From Mangroves of Guangxi, China

**DOI:** 10.1002/ece3.73695

**Published:** 2026-05-19

**Authors:** Zhaojun Zeng, Jinyi Wu, Xinlu Wu, Manning Lei, Chenxi Fang, Baohua Zhang, Jianjun Cui, Enyi Xie

**Affiliations:** ^1^ Guangdong Ocean University Zhanjiang China; ^2^ Xiamen University Xiamen China

**Keywords:** *Laurencia* complex, morphology, *Palisada yatsenii* sp. Nov, phylogeny, *rbc*L*cox*1

## Abstract

Several small, unidentified specimens of the *Laurencia* complex were collected from mangrove forests in Qinzhou, Guangxi, China. To characterize these specimens, we performed detailed morphological observations and phylogenetic analyses based on chloroplast *rbc*L and mitochondrial *cox*1 gene sequences. The results revealed that these specimens represent a previously undescribed species within the genus *Palisada*. Given that the species was discovered in Xiandao Park (also known as Yat‐sen Park), we name it *P. yatsenii* sp. nov. to honor Dr. Sun Yat‐sen, the great forerunner of China's modern democratic revolution. This species is endemic to the high intertidal mangrove habitat, a niche where no known congeneric species have been previously documented—thus expanding our ecological understanding of the genus *Palisada*. Key diagnostic traits of this species include a predominantly pale‐yellow thallus, prostrate growth habit, and the formation of irregular to subcircular cushion‐shaped clumps. Phylogenetic analyses based on the *rbc*L and *cox*1 makers separately confirmed that the studied specimens form a clade within *Palisada*, with strong support recovered in the *rbc*L phylogeny. The *rbc*L and *cox*1 genetic distances between the new species and other members of the genus *Palisada* ranged from 6.3% to 9.7% and from 5.8% to 8.4%, respectively—values that exceed the minimum threshold for species delimitation in *Palisada*. The discovery of *P. yatsenii* sp. nov. underscores the gaps in our knowledge of coastal macroalgae and emphasizes the urgency of macroalgal diversity research for the sustainable management and conservation of these biological resources.

## Graphical abstract

A new red algal species, *Palisada yatsenii* sp. nov., is described from the high intertidal mangrove habitat in Qinzhou, Guangxi, China, representing the first record of Palisada in this unique niche. Second, integrative taxonomic analyses (morphology + rbcL/cox1 phylogenetics) confirm its independence, with distinct diagnostic traits (pale yellow, prostrate and cushion‐shaped thalli) and genetic distances exceeding species delimitation thresholds. Finally, this discovery expands the ecological range of Palisada and highlights the need for enhanced macroalgal diversity surveys for coastal conservation.

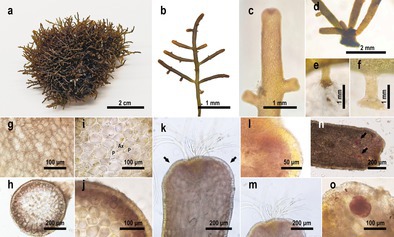

## 1 Introduction

The genus *Palisada* K.W.Nam belongs to the phylum Rhodophyta (red algae), class Florideophyceae, order Ceramiales, family Rhodomelaceae (Woelkerling et al. 2025). Prior to the widespread application of molecular biological techniques, this genus was long classified within the *Laurencia* J.V. Lamouroux complex (Nam 2007; Díaz‐Tapia et al. 2017). The establishment of *Palisada* has undergone multiple taxonomic revisions: in the 1930s, the Japanese scholar Yamada (1931) first delimited the relevant taxa as *Laurencia* sect. *Palisadae* Yamada; by the end of the 20th century, advances in morphological and anatomical studies enabled Nam et al. (1994), Garbary and Harper (1998) and Nam (1999) to split the *Laurencia* complex into three genera, namely *Laurencia*, *Chondrophycus* (J.Tokida & Y.Saito) Garbary & J.T.Harper and *Osmundea* Stackhouse, with the aforementioned taxa assigned to *Chondrophycus*; in the early 21st century, regional surveys of the *Laurencia* complex were conducted worldwide, which further accumulated morphological data for this group. Based on integrated phylogenetic analyses and morphological characteristics, the South Korean scholar Nam (2006) proposed elevating *Laurencia* sect. *Palisadae* to the generic rank and transferring several *Chondrophycus* species to this new genus. *Palisada* can be distinguished from its closely related genera by a suite of morphological features: two periaxial cells, the first periaxial cell beneath the trichoblast, absence of secondary pit connections in most species, a tetrasporangial axis with one sterile periaxial cell, and trichoblast‐type spermatangial development (Nam 2006, 2007). Subsequent studies have further verified the genetic differentiation between *Palisada* and other closely related genera, confirming the rationality of its status as an independent genus and enriching the species diversity of this genus (Díaz‐Tapia et al. 2017; Kyaw et al., 2025; Metti and Metti 2022; Woelkerling et al. 2025). To date, 26 species of *Palisada* have been taxonomically accepted (Guiry and Guiry 2026).

China's coastline spans tropical and temperate zones, with diverse habitats and abundant macroalgal resources (Liu 2013). Studies on the *Laurencia* complex in China were initiated as early as the mid‐19th century (Ding 2003). Systematic research commenced in the 1940s, when Tseng (1943) conducted taxonomic research on the *Laurencia* complex in Hong Kong, documenting five species of *Laurencia* (including four new species) now transferred to the genus *Palisada*: *L. jejuna* C.K.Tseng [≡ *P. jejuna* (C.K.Tseng) K.W.Nam], *L. paniculata* (C.Agardh) J.Agardh [= *P. thuyoides* (Kützing) Cassano, Sentíes, Gil‐Rodríguez & M.T.Fujii], *L. surculigera* C.K.Tseng [≡ *P. surculigera* (C.K.Tseng) K.W.Nam], *L. longicaulis* C.K.Tseng [≡ *P. longicaulis* (C.K.Tseng) K.W.Nam], and *L. parvipapillata* C.K.Tseng [≡ *P. parvipapillata* (C.K.Tseng) K.W.Nam]. From the late 20th century to the early 21st century, numerous scholars led by Tseng carried out surveys of marine macroalgal resources across China (Tseng 1984, 2009; Zhang 1988; Ding 2003; Xia 2013). These efforts further expanded the known distribution ranges of the aforementioned *Palisada* species and recorded four additional species: *P. capituliformis* (Yamada) K.W.Nam, *P. intermedia* (Yamada) K.W.Nam, *C. palisadus* (Yamada) K.W.Nam [= *P. robusta* K.W.Nam], and *P. papillosa* (C.Agardh) K.W.Nam [= *P. perforata* (Bory) K.W.Nam]. Actually, a total of nine *Palisada* species have been recorded in China (two of which are endemic to China), accounting for 34% of the taxonomically accepted species and indicating rich resources of this genus in the region. However, the most recent taxonomic study on *Palisada* in China (Xia 2013) is over a decade old and lacks molecular phylogenetic evidence, which may have led to ambiguities in species delineation. Additionally, previous surveys had limited geographic coverage; for example, Xia (2013) did not detect any species of *Palisada* in regions such as Guangxi, Fujian, and Jiangsu during his nationwide survey, suggesting that the species diversity in China may be underestimated.

Our research team has conducted multiple surveys of macroalgal resources in the South China Sea (Zeng et al. 2025a, 2025b). Several unidentified specimens of the *Laurencia* complex were collected from the mangrove zone of Qinzhou, Guangxi, China. Subsequent molecular and morphological identification confirmed that these specimens represent a new species of *Palisada*. In this study, we provide a detailed morphological description of this new species and clarify its phylogenetic relationships based on *rbc*L and *cox*1 gene sequences.

## 2 Materials and Methods

### 2.1 Sample Collection

**FIGURE 1:**
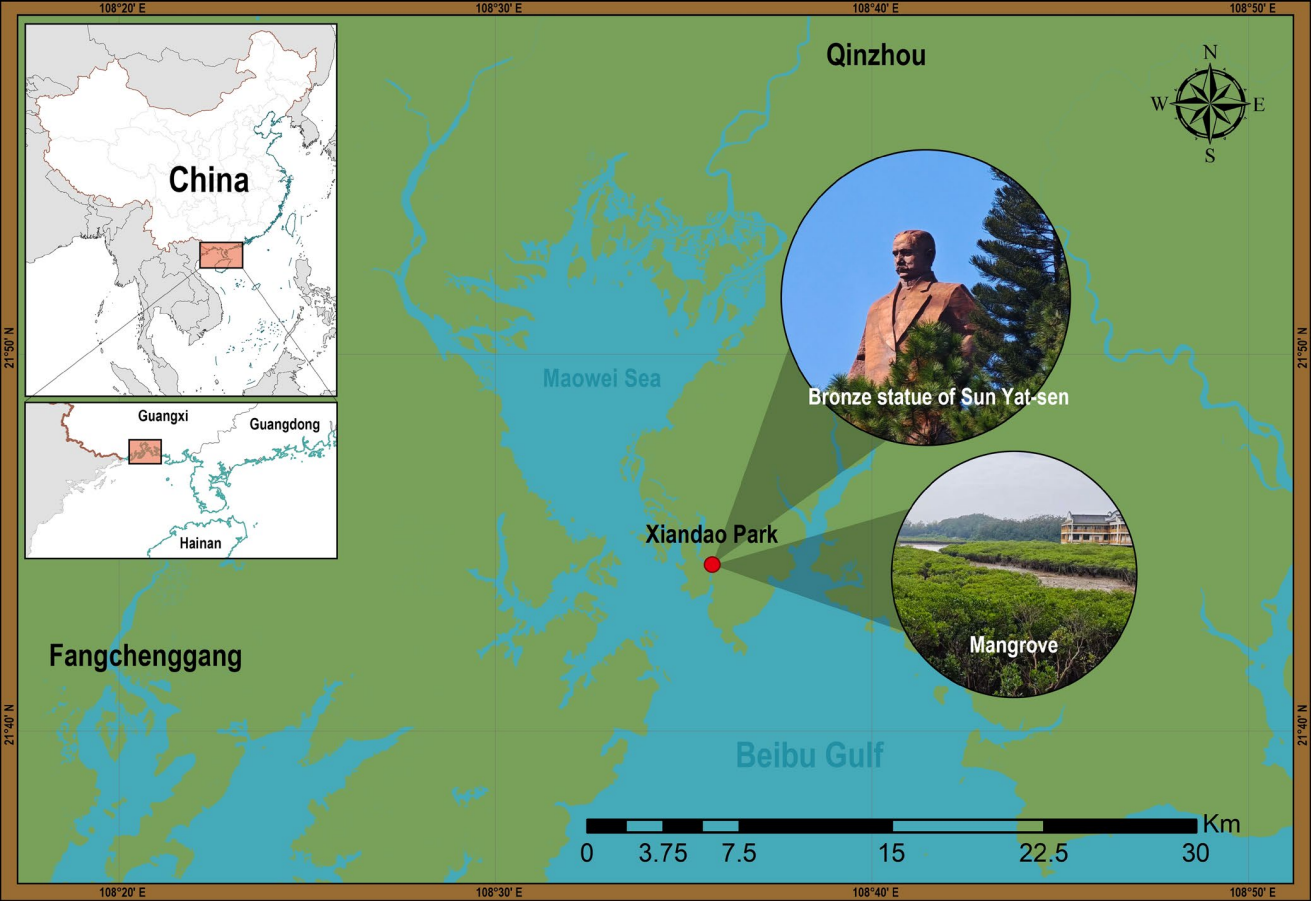
Sampling location and environment. Red dots indicate the sampling site, Xiandao Park (also called Yat‐sen Park, 21°44′28″ N, 108°35′46″ E). This park houses the world's largest bronze statue of Sun Yat‐sen and features extensive mangrove forests within its boundaries.

Specimens of *Palisada* were collected from Xiandao Park (also called Yat‐sen Park), Qinnan District, Qinzhou, Guangxi, China (21°44′28″ N, 108°35′46″ E), during two field surveys in March and December 2025 (Figure 1). The collected specimens were transported to the Laboratory of Algal Resource Development and Aquaculture Environmental Ecological Restoration, Guangdong Ocean University, within 4 h of collection. After being rinsed with sterile seawater to remove surface impurities such as gravel and epiphytic algae, the specimens were used for subsequent morphological documentation and molecular analyses.

### 2.2 Morphological Observation

Relatively intact individuals were selected, and their morphological characteristics were observed and photographed (Xiaomi 15 Pro, Xiaomi, Beijing, China) according to the methods described by Xia (2013). These characteristics included external morphological traits such as thallus shape, size, color, texture and branching pattern. Subsequently, samples were excised from representative parts of the thallus for free‐hand sectioning. The prepared glass slides were then examined under an upright optical microscope Olympus CX33 and DP74 (Olympus, Tokyo, Japan) for further observations and record internal structures, including cortical cells, medullary cells, axial cells, periaxial cells, and reproductive structures. After completing the morphological documentation, multiple herbarium specimens were prepared as voucher specimens (Table S1) and deposited in the Marine Biological Museum of the Chinese Academy of Sciences (MBMCAS), Qingdao, China (Thiers 2025); the Aquatic Organisms Museum of Guangdong Ocean University (AOMGDOU); and the Macroalgal Laboratory of Guangdong Ocean University (MLGDOU), Zhanjiang, China.

### 2.3 DNA Extraction and Amplification

Total DNA was extracted using the Plant Genomic DNA Rapid Extraction Kit (Sangon Biotech, Shanghai, China) following the manufacturer's instructions. Two gene markers widely used for the taxonomic identification of red algae globally (Nam 2006; Díaz‐Tapia et al. 2017) were selected for molecular analysis: *rbc*L and *cox*1 (Table 1). PCR amplification was performed using a Bio‐Rad C1000 Thermal Cycler. The 20 μL reaction system consisted of 1 μL DNA template (approximately 50 ng·μL^−1^), 10 μL 2 × SanTaq PCR Mix (Sangon Biotech, Shanghai, China), 1 μL each of forward and reverse primers, and 7 μL sterile double‐distilled water. The amplification program was set as follows: initial denaturation at 94°C for 3 min; 35 cycles of denaturation at 94°C for 30 s, annealing at 49.5°C for 40 s and extension at 72°C for 50 s (*cox*1) or 80 s (*rbc*L); and a final extension at 72°C for 5 min. After detection by 1% agarose gel electrophoresis, the PCR amplicons were sent to Sangon Biotech (Guangzhou, China) for cloning and sequencing. The assembled sequence data were deposited in the National Center for Biotechnology Information (NCBI, https://www.ncbi.nlm.nih.gov) database, with detailed information provided in Table S1.

**TABLE 1:** Primer information.

Gene name	Primer and sequence	References
*rbc*L	F57 (5′‐GTAATTCCATATGCTAAAATGGG –3′) R1381 (5′‐ATCTTTCCATAGATCTAAAGC –3′)	Freshwater and Rueness (1994)
*cox*1	GWSFn (5′‐TCAACAAAYCAYAAAGATATYGG –3′)	Line and Saunders (2010)
	CoxIR1 (5′‐GTATACATATGATGHGCTCAA –3′)	Saunders (2008)

### 2.4 Sequence Analyses

Sequence similarity searches were performed using BLAST in the global database NCBI, and reliable sequences from the database were selected for phylogenetic analysis by referring to existing taxonomic literature (Tables S2 and S3). For phylogenetic tree construction, *Halopithys incurva* (Hudson) Batters, *Cladurus elatus* (Sonder) Falkenberg, *Herposiphonia tenella* (C. Agardh) Ambronn, *Rhodomela confervoides* (Hudson) P.C.Silva, *Ceramium virgatum* Roth, and *Melanothamnus harveyi* (Bailey) Díaz‐Tapia & Maggs were selected as outgroups, with additional sequences of species from related genera (the *Laurencia* complex) included. MEGA v11.0.13 was used for sequence alignment, correction, and characteristic analysis (Tamura et al. 2021). Phylogenetic analyses were conducted separately for the *rbc*L and *cox*1 datasets. For each marker, the optimal evolutionary model (GTR + I + G) was selected using IQ‐TREE v3.0.1 (Wong et al. 2025), and this model was employed in MEGA v11.0.13 to construct a maximum likelihood (ML) phylogenetic tree with 1000 bootstrap replicates (Tamura et al. 2021). The GTR + I + G model was also used for Bayesian Inference (BI) in MrBayes v3.1.2 (Ronquist and Huelsenbeck 2003): 1,000,000 generations, discarding the first 25% of trees as burn‐in. Genetic distances between sequences were computed in MEGA v11.0.13 using the Kimura two‐parameter model (Tamura et al. 2021).

## 3 Results

### 3.1 *Palisada yatsenii* Z. J. Zeng and E. Y. Xie sp. nov.

**FIGURE 2:**
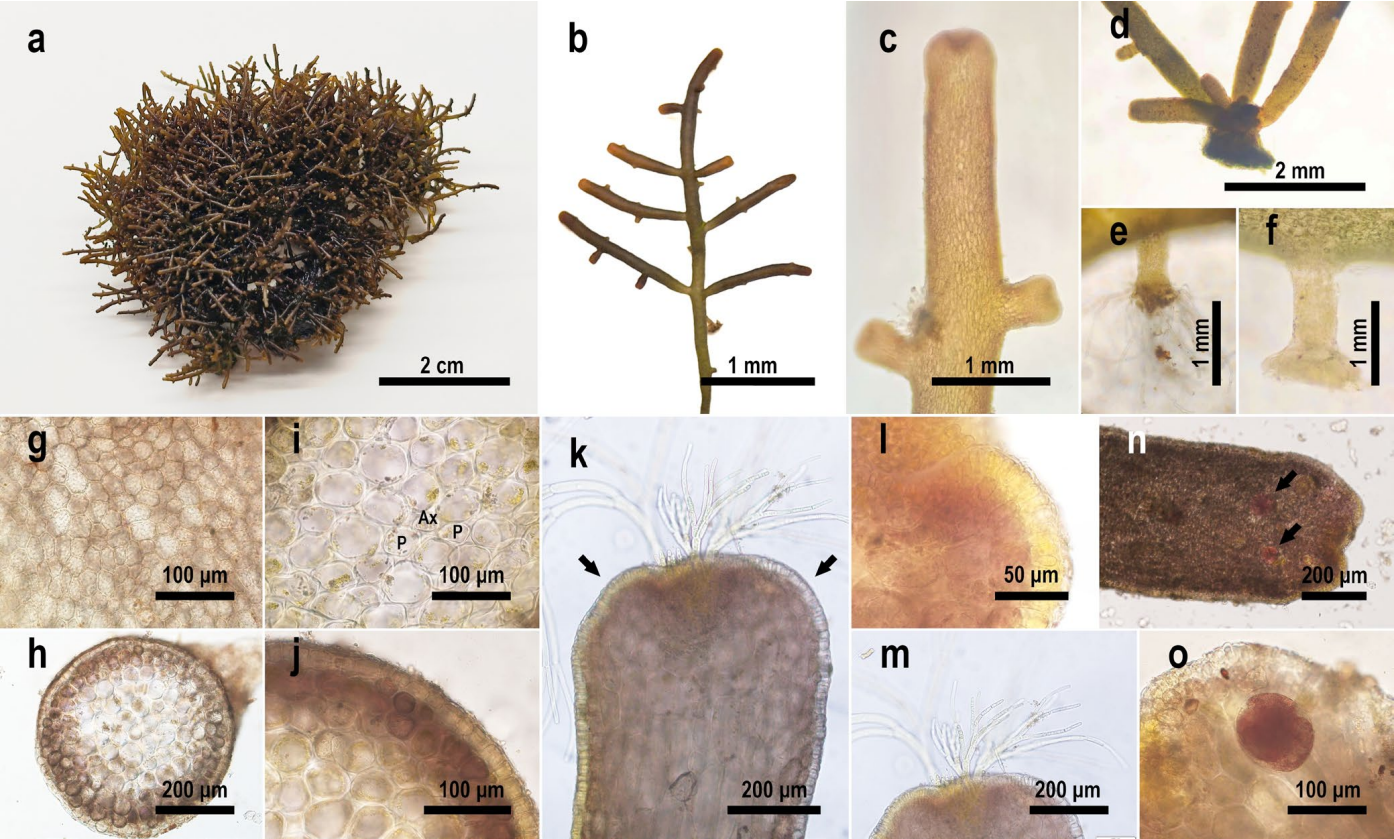
Morphology of *Palisada yatsenii* sp. nov. (a) Cushion‐shaped clumps formed by interconnected individuals; (b) single individual showing branching pattern; (c) apical portion of the thallus; (d) basal portion of the thallus with a primary discoid holdfast; (e) secondary rhizoidal holdfast; (f) secondary discoid holdfast; (g) surface view of the thallus; (h) transverse section of the thallus; (i) transverse section of the thallus, enlarged view of the medulla showing an axial cell (Ax) and two periaxial cells (P); (j) transverse section of the thallus, enlarged view of the cortex; (k) longitudinal section of the thallus, arrow indicating cortical cells with differential pigmentation on the adaxial and abaxial sides; (l) longitudinal section of the thallus apex, showing cortical cells slightly protruding beyond the thallus surface; (m) longitudinal section of the thallus apex, showing pale yellow to hyaline trichoblasts; (n) tetrasporangiate branchlet, arrow pointing to tetrasporangia; (o) tetrasporangia.

- **Diagnosis:** Thalli caespitose, prostrate, forming cushion‐shaped clumps; thallus pale yellowish‐green to brownish‐yellow, terete, to 5 cm long, cartilaginous. Branches sparse, alternate or subopposite, main branches 0.4–0.8 mm diam. Cortical cells without secondary pit connections are slightly protruding from the surface.
- **Description**: Thalli caespitose, prostrate, with multiple individuals interconnected and aggregate, irregular or subcircular cushion‐shaped clumps, less than 3 cm thick (Figure 2a). Thallus pale yellowish‐green to brownish‐yellow, mostly pale yellow, generally not exceeding 5 cm in length, cartilaginous; dried specimens weakly adherent to herbarium paper. Branches terete, often curved toward substrate, 0.4–0.8 mm in diameter, sparse, mostly alternate or subopposite, occasionally unilateral, internodes 1–5 mm; ultimate branchlets subcylindrical or clavate (Figure 2b,c). Multiple branches arising from the primary discoid holdfast at base (Figure 2d). Branches attaching to substrate or interconnecting by secondary rhizoidal and discoid holdfasts (Figure 2e,f), holdfast spacing similar to branch internodes. Fresh thalli brittle, easily broken; individuals not separable from clumps without damage. Surface cortical cells irregular, polygonal, subcircular to elongated, 10–30 μm long, 8–25 μm wide, without secondary pit connections; corps en cerise absent (Figure 2g). In transverse section, thallus cylindrical, composed of cortex and medulla (Figure 2h). Axial cells each with two periaxial cells (Figure 2i). Cortical cells subquadrate, in palisade‐like arrangement, pigment content lower than subcortical cells, 10–35 μm long, 10–30 μm wide. Subcortical cells subcircular, 20–60 μm long, 15–50 μm wide. Medullary cells subcircular, hyaline, cell walls without lenticular thickenings, 40–75 μm long, 35–65 μm wide (Figure 2j). In longitudinal section, apical meristem consistently within terminal depression (Figure 2k). Apical cortical cells slightly protruding beyond surface (Figure 2l); trichoblasts pale yellow to hyaline (Figure 2m). Cortical cells rectangular to elliptical, in palisade‐like arrangement, 15–25 μm long, 10–20 μm wide; adaxial cells pale yellow, abaxial cells hyaline. Medullary cells are oblong, 100–270 μm long, 40–70 μm wide (Figure 2k).

Tetrasporangia scattered near branch apices (Figure 2n), elliptical, cruciately divided, 80–120 μm long, 60–100 μm wide (Figure 2o). Spermatangia and cystocarps not observed.

**FIGURE 3:**
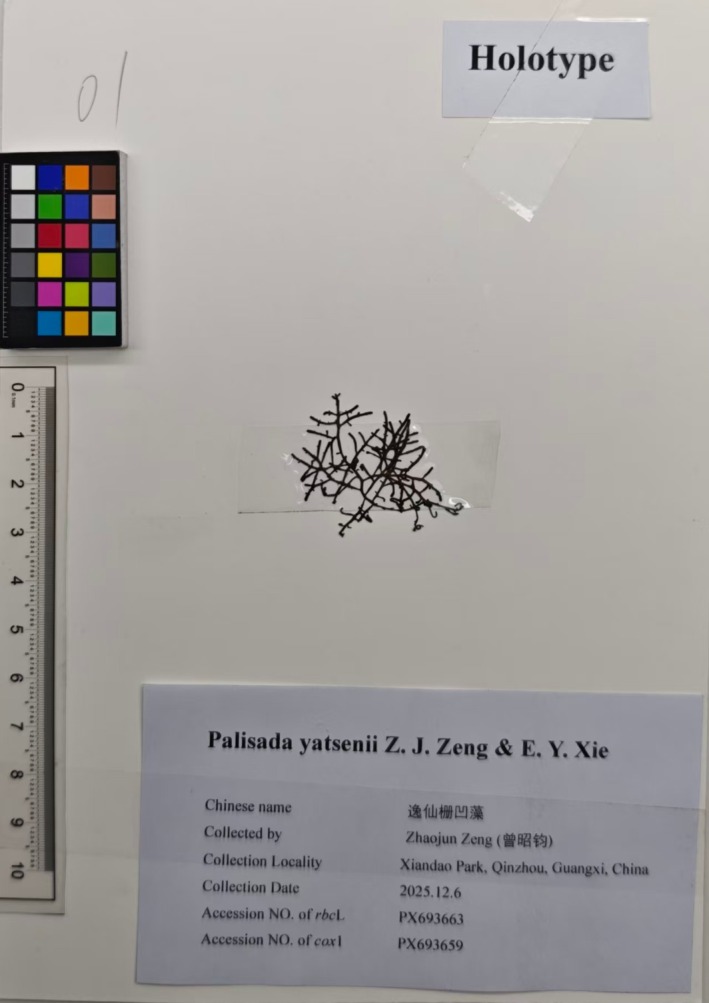
Holotype of *Palisada yatsenii* sp. nov. (MBM288563).

**FIGURE 4:**
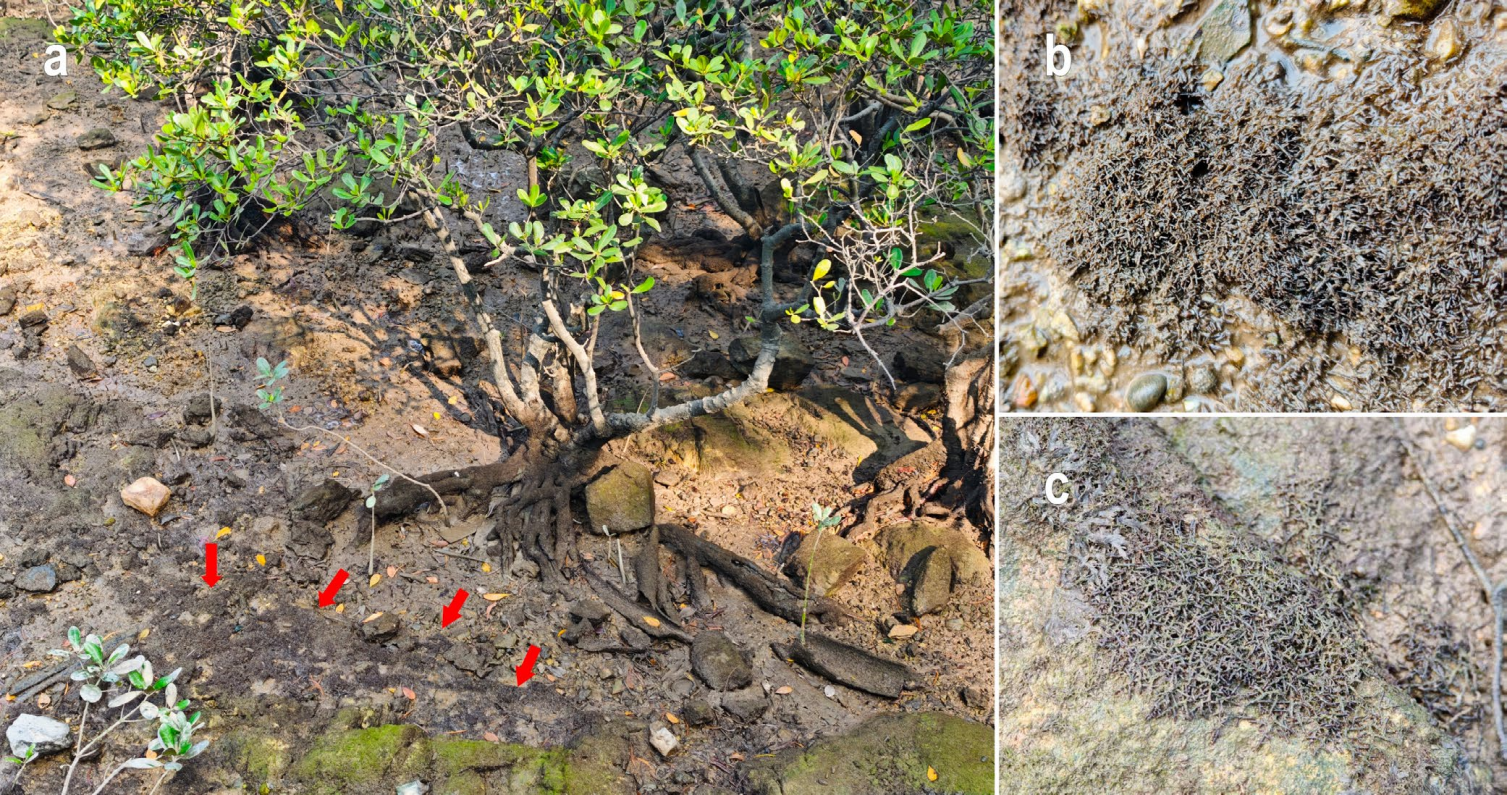
Habitat of *Palisada yatsenii* sp. nov. (a) Rock‐mud substrate in the mangrove zone of the high intertidal zone, red arrows indicate the cushion‐shaped clumps; (b) cushion‐shaped clumps adhering to gravels, with parts embedded in silt; (c) cushion‐shaped clumps attached to larger rocks.

- **Holotype**: MBM288563, Dec 6, 2025, collected by Z. Zeng; deposited in MBMCAS (Figure 3).
- **Type locality**: Xiandao Park, Qinnan District, Qinzhou, Guangxi, China (21°44′28″ N, 108°35′46″ E).
- **Isotypes**: MBM288564‐67 in MBMCAS; PL040115037D01‐02 in AOMGDOU.
- **Etymology**: The specific epithet *yatsenii* honors Dr. Sun Yat‐sen (1866–1925), the great forerunner of China's modern democratic revolution, in recognition of his extraordinary contributions to overthrowing the feudal monarchy and pioneering the democratic revolutionary movement in modern China. This species is currently only known from Xiandao Park (also called Yat‐sen Park) in Qinzhou, Guangxi, China—a park built to commemorate Sun Yat‐sen, with the world's largest bronze statue of him as its iconic landmark. Furthermore, the discovery of this species in 2025 coincides with the 100th anniversary of Sun Yat‐sen's passing, rendering the naming a fitting tribute.
- **Additional herbarium specimens**: PL040115037D03‐05 (Nov to Dec 2025) in AOMGDOU; QZ001‐003 (Nov to Dec 2025) in MLGDOU.
- **DNA sequences of type specimens**: For the holotype, PX693663 (*rbc*L) and PX693659 (*cox*1); for isotypes, PX693664–PX693665 (*rbc*L) and PX693660–PX693662 (*cox*1).
- **Distribution and habitat**: This species is currently known only from Xiandao Park, Qinzhou, Guangxi, China. It is restricted to rocky or rock‐mud substrates in the high intertidal mangrove habitat (Figure 4a) and has not been recorded on pure muddy substrates. The thalli form cushion‐shaped clumps, mostly adhering to gravels, partially embedded in silt (Figure 4b), or attached to various substrates including larger rocks, mangrove roots, and shells (Figure 4c).

### 3.2 Phylogenetic Analyses

A total of 51 sequences were used for constructing the *rbc*L phylogenetic tree, including three newly generated sequences from this study. The final aligned length of the *rbc*L sequences was 1144 bp, with 681 conserved sites (60%), 463 variable sites (40%), and 392 parsimony‐informative sites (34%). Similarly, for the *cox*1 phylogenetic tree, 41 sequences were employed, including four newly generated sequences from this study. The final aligned length of the *cox*1 sequences was 509 bp, with 308 conserved sites (61%), 201 variable sites (39%), and 168 parsimony‐informative sites (33%). The phylogenetic trees constructed using ML and BI methods exhibited similar topologies; thus, only the ML trees are presented herein, with Bayesian posterior probabilities indicated at nodes.

**FIGURE 5:**
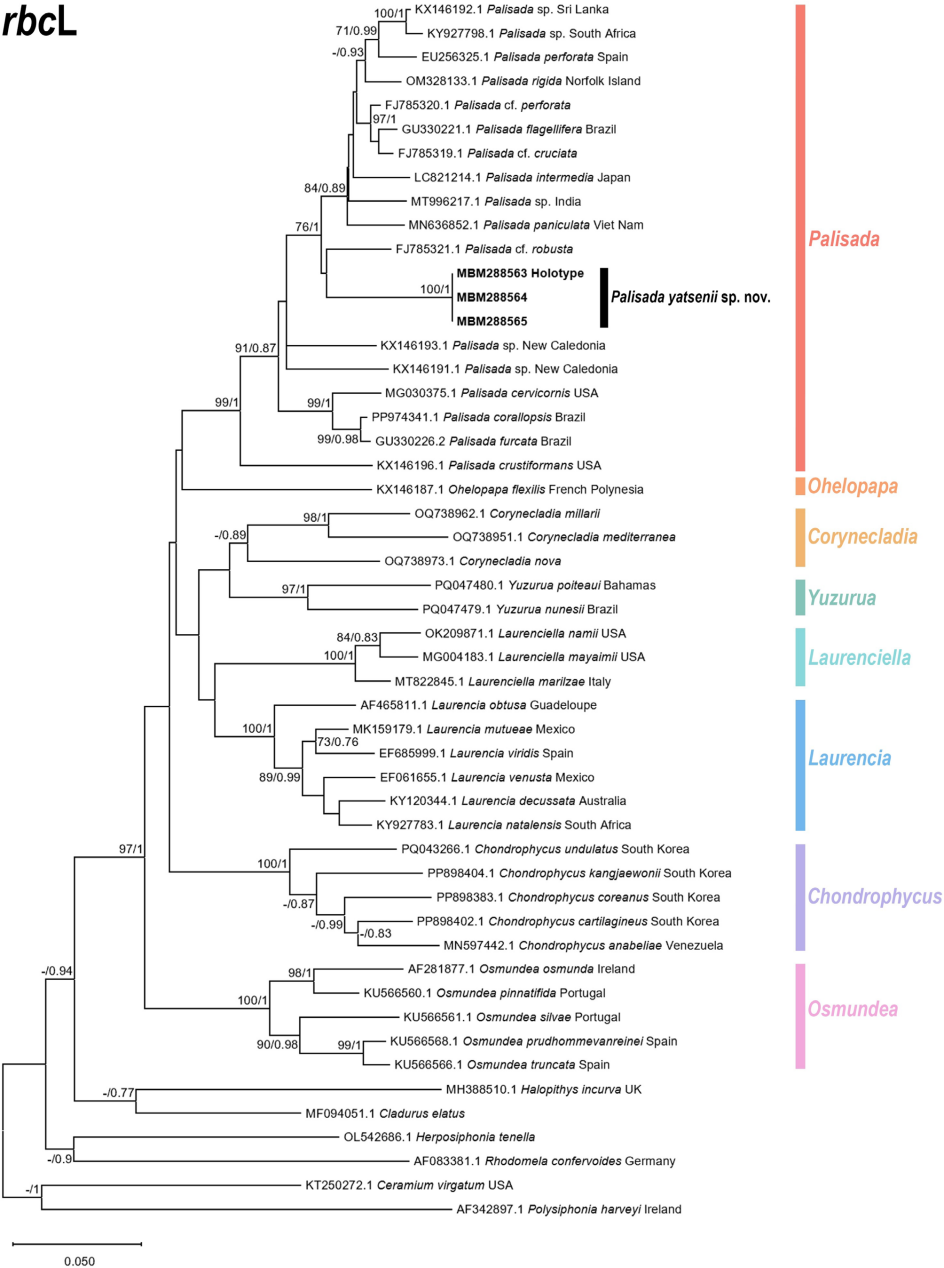
ML phylogenetic tree based on *rbc*L gene. Boldface indicates newly generated sequences generated in this study. Values adjacent to nodes represent (from left to right): Bootstrap support values for ML analyses, and posterior probabilities for BI analysis. Values are shown only for nodes with Bootstrap support ≥ 70% (ML) and posterior probability ≥ 0.7 (BI).

**FIGURE 6:**
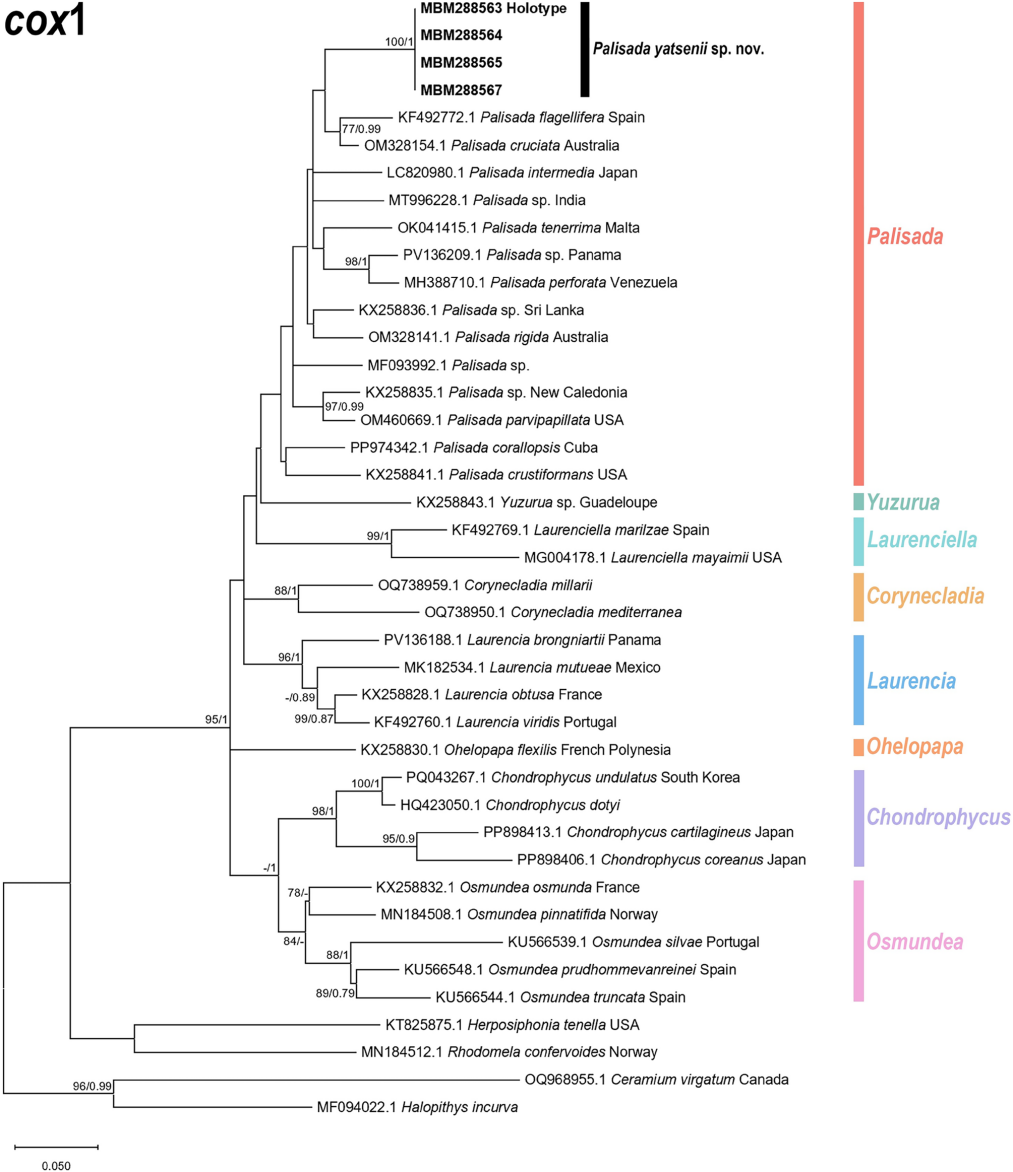
ML phylogenetic tree based on *cox*1 gene. Boldface indicates newly generated sequences generated in this study. Values adjacent to nodes represent (from left to right): Bootstrap support values for ML analyses, and posterior probabilities for BI analysis. Values are shown only for nodes with Bootstrap support ≥ 70% (ML) and posterior probability ≥ 0.7 (BI).

The results showed that in the *rbc*L phylogenetic tree (Figure 5), all *Palisada* species formed a monophyletic clade with strong bootstrap support and posterior probability (99/1) for this marker. Within this monophyletic clade, our sequences (MBM288563‐65) clustered into a single subclade with maximum phulogenetic support (100/1). No nucleotide sequence differences were detected among these three sequences, indicating they belong to the same taxon. The sequences most closely related to this taxon were from *P*. cf. *robusta* (FJ785321.1), but there was no strong bootstrap support or posterior probability for recognizing them as sister groups, and the genetic distance between the two taxa reached 6.5%. The interspecific genetic distances of the *rbc*L gene among *Palisada* species are shown in Table S4, ranging from 1.2% to 9.7%. Even the taxon with the closest genetic distance to the newly amplified sequences in this study still showed a distance of 6.3%. The *cox*1 phylogenetic tree (Figure 6) showed strong congruence with the *rbc*L phylogeny. The four newly amplified sequences (MBM288563‐65, 67) from this study clustered into a single subclade with maximum support (100/1), with no nucleotide sequence differences, confirming they belong to the same taxon. The sequences most closely related to this taxon formed a monophyletic clade consisting of *P. flagellifera* (J.Agardh) K.W.Nam (KF492772.1) and *P. cruciata* (Harvey) K.W.Nam (OM328154.1). However, this sister‐group relationship lacked strong bootstrap support and posterior probability, with genetic distances of 6.8% and 5.8% between the new taxon and *P. flagellifera* (KF492772.1) and *P. cruciata* (OM328154.1), respectively. The interspecific genetic distances of the *cox1* gene among *Palisada* species are shown in Table S5, ranging from 3.2% to 9.1%. The closest genetic distance between our sequences and any known *Palisada* taxon was 5.8%.

## 4 Discussion

Based on detailed morphological observations combined with phylogenetic analyses of chloroplast *rbc*L and mitochondrial *cox*1 sequences, the specimens collected from Qinzhou, Guangxi, China were confirmed to represent a new species, *P. yatsenii*. Since *Palisada* was long classified within the *Laurencia* complex and established as an independent genus in the early 21st century, there are currently no specific taxonomic criteria dedicated to this genus (Nam 2006). Thus, the taxonomic criteria of the *Laurencia* complex are generally adopted for taxonomic treatments (Ding 2003; Xia 2013). Unlike other macroalgae with simple morphologies, such as *Ulva* Linnaeus, the *Laurencia* complex exhibits considerable morphological diversity and possesses numerous features applicable for taxonomic identification. Approximately 70 diagnostic characteristics have been described to date (Ding 2003), and more than 20 of these characters are typically used in combination to distinguish between different species in modern taxonomic practices (Masuda et al. 1997; Kyaw and Soe‐Htun 2018; Romero‐Orozco et al. 2025).

In general, different macroalgal species exhibit distinct environmental adaptabilities, resulting in variations in their horizontal and vertical distributions (Eriksson and Bergström 2005). Species of the *Laurencia* complex are primarily distributed in a narrow zone ranging from the middle‐lower intertidal to the upper subtidal zone, with only a very small number capable of tolerating the harsh environmental conditions of the high intertidal zone. Therefore, the habitat of the *Laurencia* complex has long been regarded as a diagnostic characteristic at the species level (Ding 2003). Among the known *Palisada* species, only have been reported to grow on intertidal rocks and in tide pools (without specifying the high intertidal zone), such as *P. capituliformis*, *P. jejuna*, and *P. surculigera*; individual species have been described as inhabiting wave‐exposed intertidal rocks, for example, *P. longicaulis* (Tseng 1943; Xia 2013). In contrast, *P. yatsenii* is strictly restricted to the high intertidal mangrove habitat, being exclusively found on rocky or rock‐mud substrates and absent from purely muddy substrates. Its habitat is sheltered from wave action and subject to prolonged aerial exposure, which is distinct from that of all 26 currently known *Palisada* species—a finding that further expands our understanding of the species within this genus. Beyond habitat, *P. yatsenii* can also be readily distinguished from congeneric species by several intuitive external morphological traits, including thallus size. Tseng (1943) categorized *Palisada* species into four size classes: 1–5 cm, 6–10 cm, 11–20 cm, and > 20 cm. Fewer than 10 *Palisada* species fall into the same size class as *P. yatsenii*, and most of these can still be differentiated by distinct external features. For instance, *P. cervicornis* (Harvey) Collado‐Vides, Cassano & M.T.Fujii is characterized by prominent annular striations (Collado‐Vides et al. 2017), whereas *P. amabilis* (Yamada) K.W.Nam is erect with irregular branching patterns (Carruthers et al. 1993). Thallus color is correlated with pigment composition and is recognized as a key diagnostic characteristic at the species level (Gil‐Rodríguez and Haroun 1992). The majority of known *Palisada* species typically exhibit dark coloration; for example, among the nine species recorded in China, most are purplish‐red, with the remainder being brown, blackish‐purple, yellowish‐purple, bluish‐black, or brownish‐purple (Xia 2013). This stands in stark contrast to the pale yellowish coloration of *P. yatsenii*.

The prostrate thalli that form cushion‐shaped clumps also constitute one of the most intuitive diagnostic features of *P. yatsenii*. Most *Palisada* species typically attach to substrates via a single primary holdfast and grow in an erect form, for example, *P. robusta* ; alternatively, some species bear secondary holdfasts on the lower branches, with only the lower portions of the thalli being prostrate while the upper portions grow erect, such as *P. intermedia* (Yamada 1931; Xia 2013). Fewer than five *Palisada* species are known to possess secondary holdfasts on all branches and are capable of forming cushion‐shaped clumps. In this study, a detailed morphological comparison was conducted between *P. yatsenii* and the relatively similar species among this group (Table 2). *P. concreta* (A.B. Cribb) K.W. Nam, *P. parvipapillata*, and *P. surculigera* are all small‐sized species with prostrate thalli, secondary holdfasts, and clump‐forming growth habits, which renders them morphologically similar to *P. yatsenii* in gross morphology. Nevertheless, besides habitat and thallus color mentioned above, these species can also be readily distinguished by several intuitive external traits including thallus axial morphology, branching pattern, and main axis diameter (Agardh 1863; Yamada 1931). Whether cortical cells protrude from the thallus surface and whether secondary pit connections are present are also recognized as crucial diagnostic criteria for species within the *Laurencia* complex (Saito 1967; Ding 2003). Specifically, cortical cells do not protrude from the thallus surface in *P. concreta* and *P. surculigera*, whereas secondary pit connections are present between cortical cells in *P. parvipapillata*, which facilitates straightforward differentiation at the microanatomical level. In summary, *P. yatsenii* is characterized by distinct diagnostic traits, particularly its unique habitat; morphologically, it can be effectively distinguished from congeneric species by combining just a few intuitive features. However, like most macroalgae, morphological variability is ubiquitous among *Palisada* species (McDermid 1988). Therefore, it is imperative to further validate morphological identification results using molecular biological approaches.

**TABLE 2:** Comparisons of *Palisada yatsenii* sp. nov. with morphologically relatively similar species.

Feature	*P. yatsenii* sp. nov.	*P. concreta*	*P. parvipapillata*	*P. surculigera*
Type locality	Qinzhou, Guangxi, China	Fairfax Island, Queensland, Australia	Hongkong, China	Hongkong, China
Habitat	Mangrove zone of the high intertidal zone, rocky or rock–mud mixed substrates	Rocky surfaces from the lower intertidal zone to the upper subtidal zone on open and sheltered reef flats	Coral reefs at 0.5–1 m below the low‐tide level on reef platforms	Intertidal rocks or tide pools in concealed habitats
Growth pattern	Clumps	Clumps	Clumps	Clumps
Thallus	Terete	Terete or subterete	Terete at the base, flattened in the middle and upper portions	Terete or slightly compressed
Size	≤ 5 cm	—	~4 cm	~4.5 cm
Texture	Cartilaginous	Cartilaginous	Cartilaginous	Soft‐fleshy
Color	Pale yellowish‐green to brownish‐yellow, mostly pale yellow	Brownish‐purple	Purplish‐red	Brownish‐purple
Holdfast	Primary and secondary	Primary and secondary	Primary and secondary	Primary and secondary
Branching pattern	Sparse, mostly alternate or subopposite, occasionally unilateral	Dense, irregularly spiral	Dense, alternate, or subopposite	Sparse, subpinnate
Main branch diameter	0.4–0.8 mm	0.9–3.2 mm	1–2.5 mm	Up to 2 mm
Adhesion to paper	Slight	—	Difficult	Slight
Cortical cells (surface view)	Irregular, polygonal, subcircular, or elongated	Polygonal to elliptical	Hexagonal, elliptical to rounded polygonal	Irregular polygonal
Secondary pit connections between cortical cells	Absent	Absent	Present	Absent
Arrangement of cortical cells	Palisade	Palisade	Palisade	Palisade
Cortical cell (cross‐section view)	Subquadrate	—	Rectangular or subquadrate	Distinctly radially elongated
Cortical cell protrusion from thallus surface	Present	Absent	Present	Absent
Medullary cells (cross‐section view)	Subcircular	——	Irregular ovate or oblong‐elliptical	—
Lenticular thickenings	Absent	Absent	Absent	Absent
References	This study	Masuda and Kogame (1998)	Tseng (1943), Ding (2003), Xia (2013)	Tseng (1943), Ding (2003), Xia (2013)

*Note:* “—” stands for data not available.

In terms of molecular analyses, phylogenetic trees constructed based on the two gene markers consistently indicated that the newly amplified sequences in this study formed an independent and well‐supported lineage within the *Palisada* clade (Figures 5 and 6). For the *rbc*L and *cox*1 genes, the interspecific genetic distances among *Palisada* species ranged from 1.2% to 9.7% and 3.2% to 9.1%, respectively. Notably, the sequences showing the closest genetic distances to *P. yatsenii* were *P*. cf. *perforata* (FJ785320.1) and *P. cruciata* (OM328154.1), with genetic distances of 6.3% and 5.8%, respectively. For the relatively conserved *rbc*L gene, these values exceed the widely accepted interspecific threshold of 2% (Nam et al. 2000; Metti and Metti 2022) and are lower than the minimum intergeneric genetic distance between *Palisada* and other genera within the *Laurencia* complex (8.8%–9.1%) reported by Cassano et al. (2012). These findings further corroborate the status of *P. yatsenii* as a distinct species within the genus *Palisada*. However, it should be noted that the number of available *Palisada* species sequences in the NCBI database remains limited: the uploaded *rbc*L sequences only cover half of the currently known species, while *cox*1 sequences are even scarcer, representing fewer than one‐third of known species. Moreover, most of these sequences are not derived from samples collected at type localities. In fact, the number of sequences labeled as *Palisada* sp. or cf. in the database is even greater than that of sequences assigned to identified species. For instance, the genetic distance between *P*. cf. *perforata* (FJ785320.1) and *P. perforata* (EU256325.1) reaches 2.9%, which reflects morphological similarity between different species—a phenomenon that is not uncommon. Additionally, in the phylogenetic trees of both gene markers, the sister group of *P. yatsenii* lacks strong bootstrap support and posterior probability values. All the above phenomena have indicated that global molecular research on *Palisada* remains insufficient. A large number of species cannot be identified solely based on morphological characteristics, and it is particularly urgent to supplement sequences from type localities of each species to facilitate the identification of morphologically similar taxa. The species diversity of this genus is undoubtedly underestimated. In future studies, more in‐depth investigations should be conducted, and efforts should be made to discover new taxa closely related to *P. yatsenii*, so as to fill the phylogenetic gaps and further clarify the species boundaries within the genus *Palisada*.

Our research team has conducted multiple macroalgal resource surveys along the southern coast of China, covering several provinces including Guangxi, Guangdong, and Hainan, and encompassing diverse habitats such as mangrove forests, rocky intertidal zones, muddy intertidal zones, and seagrass beds (Zeng et al. 2025a, 2025b). However, *P. yatsenii* has only been discovered in a single mangrove forest in Qinzhou, Guangxi. This new species is strictly associated with the high intertidal mangrove zone, characterized by extreme fluctuations in desiccation, salinity, and siltation, yet it has not been found in adjacent mangrove areas. Whether this pattern represents true regional endemism or distributional restriction driven by habitat specificity requires further investigation. Including the genus *Palisada*, the entire *Laurencia* complex currently comprises 223 species, representing a species‐rich taxon (Guiry and Guiry, 2025). As primary producers and habitat formers, these species constitute an essential component of coastal marine ecosystems (Mejia et al. 2012). In addition, similar to common economically important macroalgae such as *Ulva*, *Gelidium* J.V. Lamouroux, and *Gracilaria* Greville, species within the *Laurencia* complex also exhibit potential applications in food, agar extraction, and pharmaceutical industries (Harizani et al. 2016; de Morais Martins et al. 2025). In recent years, with the acceleration of human activities such as urbanization and coastal development, the natural habitats of numerous macroalgal species have been altered, which in turn has led to a decline in their diversity and biomass (Gao et al. 2025). Species with restricted distributions, such as *P. yatsenii*, are the most vulnerable to such impacts, and gaining insights into and implementing conservation measures for these taxa are therefore extremely urgent. Continuous surveys of macroalgal species are not only conducive to the expansion of ecological knowledge and the sustainable utilization of these biological resources but also crucial for habitat conservation and the enrichment of species diversity.

## Author Contributions

**Zhaojun Zeng:** data curation (lead), formal analysis (lead), investigation (lead), methodology (lead), software (lead), visualization (lead), writing – original draft (lead). **Jinyi Wu:** investigation (lead). **Xinlu Wu:** investigation (lead). **Manning Lei:** investigation (lead). **Chenxi Fang:** investigation (supporting). **Baohua Zhang:** investigation (supporting). **Jianjun Cui:** investigation (supporting). **Enyi Xie:** conceptualization (lead), funding acquisition (lead), investigation (supporting), methodology (supporting), supervision (lead), writing – review and editing (lead).

## Funding

This research was supported by the Department of Agriculture and Rural Affairs of Guangdong Province [Grant Nos. 2024‐MRI‐001‐06 (Industrial Cultivation and Demonstration of New Macroalgal Species in Marine Ranches of Western Guangdong) and 2024‐MRI‐001‐10 (Evaluation of Carbon Sink Valuation of Macroalgae in Tidal Flat Aquaculture of Western Guangdong)].

## Ethics Statement

The authors have nothing to report.

## Conflicts of Interest

The authors declare no conflicts of interest.

## Supporting information


**Table S1:** Detailed information of the specimens collected in this study.
**Table S2:** Sample information for *rbc*L sequences from GenBank used in this study.
**Table S3:** Sample information for *cox*1 sequences from GenBank used in this study.
**Table S4:** Interspecific genetic distances of *rbc*L gene in *Palisada*.
**Table S5:** Interspecific genetic distances of *cox*1 gene in *Palisada*.

## Data Availability

The datasets generated and/or analyzed during the current study are available in the manuscript and Supporting information. Specifically, the DNA sequences generated in this study have been deposited in the NCBI database under accession numbers PX693659–PX693665. Additional details, including specimen information, GenBank sequence metadata, and interspecific genetic distances, are provided in the Supporting information.
