# Supplementary material for: Palisada yatsenii sp. nov. (Ceramiales, Rhodophyta), a New Prostrate Red Alga From Mangroves of Guangxi, China
Source: Ecol Evol. 2026 May 19;16(5):e73695. doi: 10.1002/ece3.73695 (PMC13185555; doi:10.1002/ece3.73695)
Supplement: Supplementary file 1 — Table S1: Detailed information of the specimens collected in this study. Table S2: Sample information for rbcL sequences from GenBank used in this study. Table S3: Sample information for cox1 sequences from GenBank used in this study. Table S4: Interspecific genetic distances of rbcL gene in Palisada. Table S5: Interspecific genetic distances of cox1 gene in Palisada. [file ECE3-16-e73695-s001.doc]

**TABLE S1** | Detailed information of the specimens collected in this study.

| **Strain Code** | **Depository** | **Collection Locality** | **Collection Date** | **Collector** | **Accession NO. of *rbc*L** | **Accession NO. of *cox*1** |
| --- | --- | --- | --- | --- | --- | --- |
| MBM288563 | MBMCAS | Xiandao Park, Qinzhou, Guangxi, China | 6-Dec-2025 | Zhaojun Zeng | PX693663 | PX693659 |
| MBM288564 | MBMCAS | Xiandao Park, Qinzhou, Guangxi, China | 6-Dec-2025 | Zhaojun Zeng | PX693664 | PX693660 |
| MBM288565 | MBMCAS | Xiandao Park, Qinzhou, Guangxi, China | 6-Dec-2025 | Zhaojun Zeng | PX693665 | PX693661 |
| MBM288566 | MBMCAS | Xiandao Park, Qinzhou, Guangxi, China | 6-Dec-2025 | Zhaojun Zeng | / | / |
| MBM288567 | MBMCAS | Xiandao Park, Qinzhou, Guangxi, China | 6-Dec-2025 | Zhaojun Zeng | / | PX693662 |
| PL040115037D01 | AOMGDOU | Xiandao Park, Qinzhou, Guangxi, China | 6-Dec-2025 | Zhaojun Zeng | / | / |
| PL040115037D02 | AOMGDOU | Xiandao Park, Qinzhou, Guangxi, China | 6-Dec-2025 | Zhaojun Zeng | / | / |
| PL040115037D03 | AOMGDOU | Xiandao Park, Qinzhou, Guangxi, China | 6-Nov-2025 | Zhaojun Zeng | / | / |
| PL040115037D04 | AOMGDOU | Xiandao Park, Qinzhou, Guangxi, China | 21-Dec-2025 | Zhaojun Zeng | / | / |
| PL040115037D05 | AOMGDOU | Xiandao Park, Qinzhou, Guangxi, China | 21-Dec-2025 | Zhaojun Zeng | / | / |
| QZ001 | MLGDOU | Xiandao Park, Qinzhou, Guangxi, China | 6-Dec-2025 | Zhaojun Zeng | / | / |
| QZ002 | MLGDOU | Xiandao Park, Qinzhou, Guangxi, China | 21-Dec-2025 | Zhaojun Zeng | / | / |
| QZ003 | MLGDOU | Xiandao Park, Qinzhou, Guangxi, China | 6-Nov-2025 | Zhaojun Zeng | / | / |
| “/” stands for data not available. | | | | | | |

**TABLE S2** | Sample information for *rbc*L sequences from GenBank used in this study.

| **Taxon** | **Collection Locality** | **Collection Date** | **Collector** | **Accession NO. of *rbc*L** |
| --- | --- | --- | --- | --- |
| *Ceramium virgatum* | USA: Massachusetts, Folly Cove, Gloucester, right side | 13-Apr-2010 | B. Clarkston, A. Savoie | KT250272 |
| *Halopithys incurva* | Spain: Canary Islands, Tenerife, Punta del Hidalgo | 16-May-2014 | M. Sanson | MH388510 |
| *Cladurus elatus* | / | / | / | MF094051 |
| *Herposiphonia tenella* | Portugal: Azores | / | / | OL542686 |
| *Rhodomela confervoides* | Germany: Kiel Bight | / | / | AF083381 |
| *Polysiphonia harveyi* | Ireland: Maghery | / | / | AF342897 |
| *Palisada* cf. *perforata* | / | / | / | FJ785320 |
| *Palisada flagellifera* | Brazil: Areias Negras, Rio das Ostras, Rio de Janeiro | 2005 | V. Cassano & M.B. Barros-Barreto | GU330221 |
| *Palisada* sp. | Sri Lanka: Polhena Beach | 16-Aug-2006 | Coppejans, Eric | KX146192 |
| *Palisada* sp. | South Africa | 09-Sep-2010 | JJ Bolton, RJ Anderson & CM Francis | KY927798 |
| *Palisada intermedia* | Japan: Hyogo Pref., Sumoto City, Yura, Cape Oishizaki | 2020-06-24 | Masahiro Suzuki | LC821214 |
| *Palisada* sp. | India | / | / | MT996217 |
| *Palisada* cf. *cruciata* | / | / | / | FJ785319 |
| *Palisada paniculata* | Viet Nam: Ly Son island, Quang Ngai province | / | / | MN636852 |
| *Palisada* cf. *robusta* | / | / | / | FJ785321 |
| *Palisada* sp. | New Caledonia: New Caledonia, New Caledonia, Koumac | 01-Jan-2004 | Payri, C. | KX146193 |
| *Palisada cervicornis* | USA: Florida, Key Largo, Pickles Reef | 14-Aug-2013 | Alain Duran | MG030375 |
| *Palisada* sp. | New Caledonia: Isle of Pines, New Caledonia | 01-Jan-2005 | Payri, C. | KX146191 |
| *Palisada corallopsis* | Norfolk Island: Kingston Lagoon, Slaughter Bay | 15-Mar-2005 | Y. Metti and A.J.K. Millar | PP974341 |
| *Palisada furcata* | Brazil: Praia de Tambau, Paraiba | 24-Feb-2004 | M.T. Fujii | GU330226 |
| *Palisada rigida* | Norfolk Island: Kingston Lagoon, Slaughter Bay | 15-Mar-2005 | Y. Metti and A.J.K. Millar | OM328133 |
| *Palisada perforata* | Spain: Canary Islands, Tenerife, Playa Paraiso | 14-Jul-2006 | M.C. Gil-Rodriguez, A. Senties, G. & M.T.Fujii | EU256325 |
| *Palisada crustiformans* | USA: Hawaii, Oahu, Makapuu | 26-May-2007 | Kurihara, A. | KX146196 |
| *Corynecladia nova* | / | / | / | OQ738973 |
| *Corynecladia millarii* | / | / | / | OQ738962 |
| *Corynecladia mediterranea* | / | / | / | OQ738951 |
| *Chondrophycus cartilagineus* | South Korea | 28-Apr-2023 | T.O. Cho, B.Y. Won | PP898402 |
| *Chondrophycus undulatus* | South Korea | 16-Apr-2022 | T.O. Cho, B.Y. Won | PQ043266 |
| *Chondrophycus kangjaewonii* | South Korea | 23-Apr-2022 | T.O. Cho, B.Y. Won | PP898404 |
| *Chondrophycus coreanus* | South Korea | 12-Oct-2023 | T.O. Cho, B.Y. Won | PP898383 |
| *Chondrophycus anabeliae* | Venezuela: Estado Falcon, Parque Nacional Morrocoy, Cayo Muerto | 19-May-2015 | / | MN597442 |
| *Laurencia obtusa* | Guadeloupe: Pointe de la Verdure | / | / | AF465811 |
| *Laurencia venusta* | Mexico: Punta Brava, Puerto Morelos, Quintana Roo | 2004 | Jhoana Diaz Larrea & Abel Senties Granados | EF061655 |
| *Laurencia mutueae* | Mexico: Acapulco, La Roqueta | / | Abel Senties | MK159179 |
| *Laurencia decussata* | Australia: NSW, Arrawarra headland | 28-Jul-2004 | Yola Metti | KY120344 |
| *Laurencia viridis* | Spain: Roca Negra Punta del Hidalgo Tenerife-Islas Canarias | 06-Oct-2005 | Gil-Rodriguez | EF685999 |
| *Laurencia natalensis* | South Africa | 19-Mar-2011 | JJ Bolton, RJ Anderson & CM Francis | KY927783 |
| *Laurenciella marilzae* | Italy: Ustica Island, Palermo | 04-Sep-2017 | D.Serio, G.Furnari et Y.Metti | MT822845 |
| *Laurenciella namii* | USA: Florida Keys | 29-May-2013 | C. Schneider, C. Lane, D. McDevit, T. Popolizio | OK209871 |
| *Laurenciella mayaimii* | USA: Florida, Key Largo | 14-Aug-2013 | / | MG004183 |
| *Ohelopapa flexilis* | French Polynesia: Tahiti, Tahiti, Tahara reef | 24-Mar-2007 | Apham, A. | KX146187 |
| *Osmundea osmunda* | Ireland | / | / | AF281877 |
| *Osmundea prudhommevanreinei* | Spain: Canary Islands, Tenerife, Playa Paraiso | 08-May-2012 | M.C. Gil-Rodriguez & M. Machin-Sanchez | KU566568 |
| *Osmundea truncata* | Spain: Canary Islands, Tenerife, El Pris | 07-May-2012 | M. Machin-Sanchez | KU566566 |
| *Osmundea silvae* | Portugal: Madeira, Madeira, Porto da Cruz | 06-Jul-2011 | M.T. Fujii, A. Neto, H. Encarnacao & M. Machin-Sanchez | KU566561 |
| *Osmundea pinnatifida* | Portugal: Madeira, Madeira, Porto da Cruz | 06-Jul-2011 | M.T. Fujii, A. Neto, H. Encarnacao & M. Machin-Sanchez | KU566560 |
| *Yuzurua poiteaui* | Bahamas: Paradise Island, Cabbage Beach | 07-Jul-2018 | B. Brunelli | PQ047480 |
| *Yuzurua nunesii* | Brazil: Bahia, Barra Grande, Marau Peninsula | 06-Jul-2019 | M. Fujii | PQ047479 |
| “/” stands for data not available. | | | | |

**TABLE S3** | Sample information for *cox*1 sequences from GenBank used in this study.

| **Taxon** | **Collection Locality** | **Collection Date** | **Collector** | **Accession NO. of *cox*1** |
| --- | --- | --- | --- | --- |
| *Ceramium virgatum* | Canada: Nova Scotia, Meisners Beach, Mahone Bay | 13-Aug-2012 | G.W. Saunders, A. Savoie, M. Bruce, K. Dixon & C. Longtin | OQ968955 |
| *Halopithys incurva* | / | / | / | MF094022 |
| *Herposiphonia tenella* | USA: Beaufort, North Carolina | 26-Oct-2003 | / | KT825875 |
| *Rhodomela confervoides* | Norway: Hakonsund | 10-Jun-2016 | G.W. Saunders & T. Bringloe | MN184512 |
| *Melanothamnus harveyi* | Canada: Prince Edward Island, Anglo Rustico | 09-Aug-2016 | G.W. Saunders, T. Bringloe, C. Brooks & A. Savoie | PP866067 |
| *Palisada* sp. | Japan: Hyogo Pref., Sumoto City, Yura, Cape Oishizaki | 2020-06-24 | Masahiro Suzuki | MF093992 |
| *Palisada intermedia* | Japan: Hyogo Pref., Sumoto City, Yura, Cape Oishizaki | 2020-06-24 | Masahiro Suzuki | LC820980 |
| *Palisada* sp. | Sri Lanka: Polhena Beach | 16-Aug-2006 | Coppejans, Eric | KX258836 |
| *Palisada* sp. | India | 12-Jan-2016 | Felix Bast | MT996228 |
| *Palisada tenerrima* | Malta | / | / | OK041415 |
| *Palisada* sp. | Panama: Bocas Del Toro, Bocas del Toro, Bocas del Toro, Beach across from STRI-Bocas Research Station | 13-Jul-2007 | Suzanne Fredericq | PV136209 |
| *Palisada* sp. | New Caledonia: Isle of Pines, New Caledonia | 01-Jan-2005 | Payri, C. | KX258835 |
| *Palisada perforata* | Venezuela: Falcon, Playa Buchuacos | 06-Oct-2012 | G. Garcia-Soto | MH388710 |
| *Palisada parvipapillata* | USA: Oahu, Kaneohe Bay, Kapaka Island, intertidal zone | 25-May-2017 | Barrett Brooks, Melinda Peters, Sarah Vasconcellos, Scott Chulakote, Seaenna Correa-Garcia, Lindsay Tanabe, Laurie Penland, Nicole Yamase | OM460669 |
| *Palisada corallopsis* | Cuba: La Habana, Rincon de Guanabo | 20-Feb-2020 | A. Areces & D. Reyes | PP974342 |
| *Palisada flagellifera* | Spain: Canary Islands, Tenerife, Punta del Hidalgo | 13-Jan-2012 | Ma Candelaria Gil Rodriguez, Maria Machin Sanchez | KF492772 |
| *Palisada cruciata* | Australia: NSW, Sydney, La Perouse, Bare Island | 29-Aug-2004 | Y. Metti | OM328154 |
| *Palisada rigida* | Australia: NSW, Botany Bay, Kurnell | 17-Nov-2005 | Danielle Williams | OM328141 |
| *Palisada crustiformans* | USA: Hawaii, Oahu, Makapuu | 26-May-2007 | Kurihara, A. | KX258841 |
| *Corynecladia millarii* | / | / | / | OQ738959 |
| *Corynecladia mediterranea* | / | / | / | OQ738950 |
| *Corynecladia elata* | / | 15-Nov-2008 | J.Eu | OR046643 |
| *Chondrophycus kangjaewonii* | South Korea | 23-Apr-2022 | T.O. Cho, B.Y. Won | PP898414 |
| *Chondrophycus undulatus* | South Korea | 16-Apr-2022 | T.O. Cho, B.Y. Won | PQ043267 |
| *Chondrophycus cartilagineus* | Japan | 23-Apr-2008 | T.O. Cho | PP898413 |
| *Chondrophycus dotyi* | / | / | / | HQ423050 |
| *Chondrophycus coreanus* | Japan | 23-Apr-2008 | T.O. Cho | PP898406 |
| *Laurencia obtusa* | France: Languedoc-Roussillon, Pyrenees-Orientales, Cap Beart, Banyuls-sur-Mer | 11-Jul-2007 | Bittner, L. | KX258828 |
| *Laurencia clavata* | Australia: Victoria, Point Lonsdale, town beach | 18-May-2010 | G.T.Kraft, R.Kraft & B.Dog jr | MK260194 |
| *Laurencia mutueae* | Mexico: Acapulco, La Roqueta | / | Abel Senties | MK182534 |
| *Laurencia viridis* | Portugal: Azores, Santa Maria, Boca de ribeira seca | 02-Jul-2011 | Mutue Toyota Fujii, Ana Neto, Joana Pombo,Maria Machin Sanchez | KF492760 |
| *Laurencia brongniartii* | Panama: Bocas Del Toro, Bocas Del Toro, Bocas Del Toro, Beach across from STRI-Bocas Research  Station, Playa Istmito, Isla Colon | 18-Jul-2007 | Suzanne Fredericq | PV136188 |
| *Laurenciella marilzae* | Spain: Canary Islands, Tenerife, Punta del Hidalgo | 13-Jan-2012 | Ma Candelaria Gil Rodriguez, Maria Machin Sanchez | KF492769 |
| *Laurenciella mayaimii* | USA: Florida, Key Largo | 14-Aug-2013 | / | MG004178 |
| *Ohelopapa flexilis* | French Polynesia: Tahiti, Tahiti, Tahara reef | 24-Mar-2007 | Apham, A. | KX258830 |
| *Osmundea osmunda* | France: Brittany, Finistere, Roscoff | 12-May-2002 | Rousseau, F. | KX258832 |
| *Osmundea prudhommevanreinei* | Spain: Canary Islands, Tenerife, Playa Paraiso | 04-May-2011 | M.C. Gil-Rodriguez & M. Machin-Sanchez | KU566548 |
| *Osmundea truncata* | Spain: Canary Islands, Tenerife, El Pris | 07-May-2012 | M. Machin-Sanchez | KU566544 |
| *Osmundea silvae* | Portugal: Madeira, Madeira, Sao Jorge | 06-Jul-2011 | E. Nogueira, V. Cassano & A. Senties | KU566539 |
| *Osmundea pinnatifida* | Norway: Hordaland, Stora Kalsoy | 19-Apr-2016 | P. Hribovesek, H. Sundahl | MN184508 |
| *Yuzurua poiteaui* | USA: Florida Keys | 29-May-2013 | C. Schneider, C. Lane, D. McDevit, T | OK209895 |
| *Yuzurua* sp. | Guadeloupe: Antilles, Carribean, Chenal ilet Colas, Grand Cul-de-Sac Marin | 03-May-2012 | Line Le Gall | KX258843 |
| “/” stands for data not available. | | | | |

**TABLE S4** | Interspecific genetic distances of *rbc*L gene in *Palisada.*

|  | 1 | 2 | 3 | 4 | 5 | 6 | 7 | 8 | 9 | 10 | 11 | 12 | 13 | 14 | 15 | 16 | 17 |
| --- | --- | --- | --- | --- | --- | --- | --- | --- | --- | --- | --- | --- | --- | --- | --- | --- | --- |
| 1. *P. yatsenii* sp. nov. |  |  |  |  |  |  |  |  |  |  |  |  |  |  |  |  |  |
| 2. FJ785320.1 *P.* cf. *perforata* | 6.3 |  |  |  |  |  |  |  |  |  |  |  |  |  |  |  |  |
| 3. GU330221.1 *P. flagellifera* | 6.4 | 1.4 |  |  |  |  |  |  |  |  |  |  |  |  |  |  |  |
| 4. FJ785321.1 *P.* cf. *robusta* | 6.5 | 4.6 | 4.9 |  |  |  |  |  |  |  |  |  |  |  |  |  |  |
| 5. KX146192.1 *Palisada* sp. | 6.5 | 3 | 3.3 | 5.8 |  |  |  |  |  |  |  |  |  |  |  |  |  |
| 6. FJ785319.1 *P.* cf. *cruciata* | 6.5 | 1.2 | 1.3 | 4.7 | 3.4 |  |  |  |  |  |  |  |  |  |  |  |  |
| 7. LC821214.1 *P. intermedia* | 6.6 | 3.1 | 3.4 | 5.3 | 4 | 3.3 |  |  |  |  |  |  |  |  |  |  |  |
| 8. MT996217.1 *Palisada* sp. | 6.6 | 3.3 | 3.4 | 5.4 | 4.3 | 3.5 | 4.1 |  |  |  |  |  |  |  |  |  |  |
| 9. KY927798.1 *Palisada* sp. | 6.8 | 3.5 | 3.7 | 6 | 0.9 | 3.8 | 4.2 | 4.6 |  |  |  |  |  |  |  |  |  |
| 10. MN636852.1 *P. paniculata* | 6.9 | 3.2 | 3.6 | 5.2 | 4.4 | 3.5 | 4.6 | 3.8 | 4.9 |  |  |  |  |  |  |  |  |
| 11. OM328133.1 *P. rigida* | 6.9 | 2.7 | 2.7 | 4.4 | 3 | 3 | 3.6 | 4 | 3 | 3.6 |  |  |  |  |  |  |  |
| 12. EU256325.1 *P. perforata* | 7.3 | 2.9 | 3.2 | 5.2 | 2.1 | 3.5 | 4.7 | 4.3 | 3.2 | 4.2 | 3 |  |  |  |  |  |  |
| 13. GU330226.2 *P. furcata* | 7.6 | 5.4 | 6 | 5.4 | 7.1 | 5.5 | 6.9 | 6.3 | 7.6 | 5.5 | 6.2 | 6.3 |  |  |  |  |  |
| 14. KX146191.1 *Palisada* sp. | 7.7 | 5.5 | 5.6 | 6.9 | 6 | 5.5 | 6.3 | 6 | 6.5 | 6 | 5.9 | 6.7 | 6 |  |  |  |  |
| 15. KX146193.1 *Palisada* sp. | 7.8 | 5.5 | 5.4 | 6.1 | 6 | 5.5 | 7.1 | 6.3 | 6.5 | 5.5 | 5.6 | 5.7 | 5.9 | 6.2 |  |  |  |
| 16. PP974341.1 *P. corallopsis* | 7.8 | 5.6 | 6.2 | 5.4 | 7.3 | 5.7 | 7.2 | 6.6 | 7.8 | 5.8 | 6.7 | 6.8 | 0.7 | 6 | 5.9 |  |  |
| 17. MG030375.1 *P. cervicornis* | 8.1 | 6.2 | 6.6 | 6.1 | 6.7 | 6.4 | 7.2 | 7.1 | 7.2 | 6.7 | 6.8 | 6.2 | 3.3 | 6.7 | 6.1 | 3 |  |
| 18. KX146196.1 *P. crustiformans* | 9.7 | 8.1 | 7.4 | 7.8 | 7.9 | 7.7 | 8.4 | 7.7 | 8.7 | 7.8 | 8.3 | 7.4 | 7.9 | 7.6 | 8.1 | 7.7 | 7.3 |

**TABLE S5** | Interspecific genetic distances of *cox*1 gene in *Palisada*.

|  | 1 | 2 | 3 | 4 | 5 | 6 | 7 | 8 | 9 | 10 | 11 | 12 | 13 | 14 |
| --- | --- | --- | --- | --- | --- | --- | --- | --- | --- | --- | --- | --- | --- | --- |
| 1. *P. yatsenii* sp. nov. |  |  |  |  |  |  |  |  |  |  |  |  |  |  |
| 2. OM328154.1 *P. cruciata* | 5.8 |  |  |  |  |  |  |  |  |  |  |  |  |  |
| 3. KX258836.1 *Palisada* sp. | 6.2 | 4.9 |  |  |  |  |  |  |  |  |  |  |  |  |
| 4. KF492772.1 *P. flagellifera* | 6.9 | 3.7 | 5.3 |  |  |  |  |  |  |  |  |  |  |  |
| 5. OM328141.1 *P. rigida* | 7.1 | 5.1 | 4.3 | 6 |  |  |  |  |  |  |  |  |  |  |
| 6. MT996228.1 *Palisada* sp. | 7.3 | 5.5 | 5.3 | 5.7 | 6 |  |  |  |  |  |  |  |  |  |
| 7. LC820980.1 *P. intermedia* | 7.5 | 5.1 | 5.3 | 6.4 | 6 | 6.6 |  |  |  |  |  |  |  |  |
| 8. OK041415.1 *P. tenerrima* | 7.6 | 6.2 | 5.8 | 6.2 | 5.8 | 6.4 | 7.3 |  |  |  |  |  |  |  |
| 9. PV136209.1 *Palisada* sp. | 7.8 | 6.2 | 5.6 | 6.7 | 5.4 | 6.6 | 7.1 | 6 |  |  |  |  |  |  |
| 10. MH388710.1 *P. perforata* | 7.8 | 5.4 | 5.8 | 6.9 | 6.5 | 6.6 | 7.6 | 7.1 | 3.2 |  |  |  |  |  |
| 11. MF093992.1 *Palisada* sp. | 8.2 | 6.2 | 5.8 | 7.3 | 6.4 | 7.1 | 6.6 | 6.9 | 6.7 | 7.8 |  |  |  |  |
| 12. KX258835.1 *Palisada* sp. | 8.2 | 6.4 | 6 | 7.1 | 6 | 6.8 | 7.3 | 7.1 | 6.6 | 6.6 | 6.4 |  |  |  |
| 13. OM460669.1 *P. parvipapillata* | 8.4 | 6 | 6 | 6.6 | 6.8 | 6.6 | 6.8 | 7.5 | 7.1 | 7.1 | 6.2 | 3.5 |  |  |
| 14. PP974342.1 *P. corallopsis* | 8.4 | 6.8 | 6 | 6.8 | 6.8 | 6.4 | 7 | 8.2 | 7.7 | 8.2 | 6.6 | 6.2 | 6.2 |  |
| 15. KX258841.1 *P. crustiformans* | 8.4 | 7.5 | 6 | 8.6 | 7 | 7.9 | 8.4 | 8.9 | 8.8 | 9.1 | 7.3 | 6.6 | 6.8 | 6.2 |
